# TiO_2_-Based Photocatalytic Geopolymers for Nitric Oxide Degradation

**DOI:** 10.3390/ma9070513

**Published:** 2016-06-24

**Authors:** Alberto Strini, Giuseppina Roviello, Laura Ricciotti, Claudio Ferone, Francesco Messina, Luca Schiavi, Davide Corsaro, Raffaele Cioffi

**Affiliations:** 1 Construction Technologies Institute-National Research Council of Italy (ITC-CNR), via Lombardia 49, San Giuliano Milanese I-20098, Italy; luca.schiavi@itc.cnr.it (L.S.); davide.corsaro@itc.cnr.it (D.C.); 2 Department of Engineering, University of Naples “Parthenope”, INSTM Research Unit Napoli Parthenope, Centro Direzionale Isola C4, Naples I-80143, Italy; laura.ricciotti@uniparthenope.it (L.R.); claudio.ferone@uniparthenope.it (C.F.); francesco.messina@uniparthenope.it (F.M.); raffaele.cioffi@uniparthenope.it (R.C.); 3 INSTM, Consorzio Interuniversitario per la Scienza e Tecnologia dei Materiali, Via G. Giusti, 9, Firenze I-50121, Italy

**Keywords:** geopolymer, alkali activated material, photocatalysis, metakaolin, fly ash, titanium dioxide

## Abstract

This study presents an experimental overview for the development of photocatalytic materials based on geopolymer binders as catalyst support matrices. Particularly, geopolymer matrices obtained from different solid precursors (fly ash and metakaolin), composite systems (siloxane-hybrid, foamed hybrid), and curing temperatures (room temperature and 60 °C) were investigated for the same photocatalyst content (i.e., 3% TiO_2_ by weight of paste). The geopolymer matrices were previously designed for different applications, ranging from insulating (foam) to structural materials. The photocatalytic activity was evaluated as NO degradation in air, and the results were compared with an ordinary Portland cement reference. The studied matrices demonstrated highly variable photocatalytic performance depending on both matrix constituents and the curing temperature, with promising activity revealed by the geopolymers based on fly ash and metakaolin. Furthermore, microstructural features and titania dispersion in the matrices were assessed by scanning electron microscopy (SEM) and energy dispersive X-ray (EDS) analyses. Particularly, EDS analyses of sample sections indicated segregation effects of titania in the surface layer, with consequent enhancement or depletion of the catalyst concentration in the active sample region, suggesting non-negligible transport phenomena during the curing process. The described results demonstrated that geopolymer binders can be interesting catalyst support matrices for the development of photocatalytic materials and indicated a large potential for the exploitation of their peculiar features.

## 1. Introduction

The photocatalytic oxidation (PCO) technology gained great attention in recent years thanks to the possible applications in both energy production (e.g., hydrogen generation by water splitting [1] or photovoltaic generation with Graetzel cells [2]) and pollution control (as advanced oxidation process for polluted air [3] and water [4] treatment). Ambient applications typically involve the development of photocatalytic devices for air or water active treatment or photocatalytic materials to be placed in the target environment with large surface installations. This latter application needs the development of viable materials typically obtained by dispersing a photocatalyst into a suitable matrix that must, at the same time, secure the catalyst and allow the exchange of reacting species with the operating environment. Cement-based matrices are widely studied at both laboratory [5,6] and field scale [7,8] in order to develop photocatalytic finishing materials for building and environmental applications with depolluting [9] and self-cleaning [10,11,12,13] properties.

Cement, particularly cement clinker production, represents an environmental issue in terms of CO_2_ emissions, accounting for 5%–8% of global CO_2_ production [14]. Several strategies have been highlighted for the reduction of embedded CO_2_ in built environments [15]. Apart from plant efficiency and process upgrades and updates, alternative sustainable binders have been proposed in the literature. The main category is represented by binders based on alkali-activated materials (AAMs) and, particularly, geopolymers that can be synthesized by means of alkaline activation of several solid precursors (e.g., fly ash [16,17,18] or calcined clays [19,20,21]), allowing more sustainable processes than traditional clinker production. Starting from the basis of geopolymers, hybrid organic-geopolymer/inorganic binders have been proposed in the literature. These innovative functional materials are obtained by the in situ co-reticulation of metakaolin, a mixture of dialkylsiloxane oligomers with different degrees of polymerization and an alkaline solution. These hybrid materials, despite the small amount of contained siloxanes, are characterized by highly interpenetrated structures, whose properties are not the sum of the single contributions from each phase, but derive from the synergistic interaction between the phases that arises from interfacial forces at the nanometric scales [22,23,24]. These materials revealed widely tunable performance depending on composition and preparation, with significant potential in the fields of structural [25], fire-resistant, and insulating [26,27] applications.

The use of AAMs as a catalyst support matrix for PCO applications is therefore very promising in the perspective of a general environmental footprint reduction in the built environment and considering the wider variety of AAMs in respect of cementitious matrix. Preliminary results are available in few studies. Fallah et al. [28,29] reported on the synthesis of a Cu_2_O/TiO_2_ composite photocatalyst dispersed within a metakaolin-based geopolymer matrix, which showed very effective photocatalytic activity under specific experimental conditions. Gasca-Tirado et al. [30,31] described the incorporation of titanium dioxide in a metakaolin-based geopolymer matrix by ion exchange with a titania precursor, as an alternative pathway for the preparation of photocatalytic geopolymers only in the case of intermixing procedure. In view of long-term effectiveness, geopolymers might provide more reliable photocatalytic performance due to different chemistries with respect to cementitious systems. In this regard, Chen and Poon [32] highlighted potential limitations associated to surface carbonation, which is a recurrent durability issue with cement-based composites. Furthermore, in the case of road tunnels, the possibility of relying on geopolymer binders that are able to reduce spalling phenomena is highly desirable. This promising fire-resistant feature is assessed in the literature for both neat [33] and hybrid geopolymers [26]. 

In this study, for the first time, a preliminary assessment of a wide set of photocatalytic geopolymer/hybrid binders was carried out. Different solid precursors such as metakaolin and fly ash were used. Particularly, the following matrices were considered: (i) metakaolin geopolymer (MK samples); (ii) fly ash geopolymer (FA samples); (iii) hybrid siloxane–metakaolin geopolymer (HS samples); and (iv) foamed hybrid siloxane–metakaolin geopolymer (FHS samples). For all the investigated systems, the influence of the curing temperature was evaluated. The photocatalytic degradation of NO and NO*_x_* was assessed, and microstructural characterization was carried out by means of coupled SEM-EDS analysis. The monitoring of these main features related to the wide set of binders for variable industrial applications and different curing conditions allowed the description of main trends and defined outlines for future research work. MK and FA samples were designed as binders for structural purposes, and, particularly, hybrid ones were expected to provide very wide design perspectives for structural reinforcement [25], improved strength/toughness [22], and enhanced thermal stability [26,27]. In the case of foamed materials (FHS), the main application can be found in thermal and acoustic insulation. The study was so extended to different supports for photocatalysis, even if characterized by an independent mix design and variable preparation complexities, in order to define main issues and thus better program future researches.

## 2. Experimental Section

### 2.1. Materials and Methods

Metakaolin was kindly provided by Neuchem S.r.l. (Milan, Italy), and its composition is reported in Table 1. Fly ash “EFA-Füller HP”, whose composition was reported in Table 1, was supplied by BauMineral GmbH, Herten, Germany. Sodium hydroxide with reagent grade was supplied by Sigma-Aldrich. The sodium silicate solution was supplied by Prochin Italia S.r.l. with the composition reported in Table 1. A commercial oligomeric dimethylsiloxane mixture was purchased from Globalchimica S.r.l. with the name of Globasil AL20. Silicon powder ~325 mesh was purchased from Sigma-Aldrich. Photocatalytic titanium dioxide (P25) with a specific surface area of 50 ± 15 m^2^·g^−1^ (Brunauer–Emmett–Teller) and an average particle size of 21 nm (according to the manufacturer) was supplied by Evonik Degussa.

### 2.2. Photocatalytic Specimens Preparation

All samples were prepared in glass Petri dishes (diameter 9.0 ± 0.1 cm, exposed area 63.5 ± 1 cm^2^).

#### 2.2.1. Metakaolin (MK and MK60) and Fly Ash (FA and FA60) Geopolymer-Based Samples

Concerning to MK and MK60 synthesis, the alkaline activating solution was prepared by dissolving solid sodium hydroxide into the sodium silicate solution. The solution was then allowed to equilibrate and cool for 24 h. The composition of the obtained solution can be expressed as Na_2_O·1.34SiO_2_·10.5H_2_O. Meanwhile, in the case of the preparation of FA and FA60 specimens, the activating solution was obtained by means of mixing of the sodium silicate solution with a sodium hydroxide solution (15 M). Moreover, in this case, the solution was left to equilibrate and cool for 24 h. Its composition can be expressed as Na_2_O·0.7SiO_2_·10.5H_2_O. For both sets of samples, the raw materials (metakaolin for MK and MK60 and fly ash for FA and FA60, respectively) were incorporated into the activating solution (with a liquid-to-solid ratio of 1.4:1 by weight for metakaolin-based samples and 0.66:1 for fly-ash-based samples respectively) and mixed with a mechanical mixer for 10 min at 800 rpm. Finally, the photocatalyst (3% by weight with respect to geopolymer paste) was added to the freshly prepared geopolymer suspension and quickly incorporated by controlled mixing (5 min at 1000 rpm).

#### 2.2.2. Hybrid Siloxane–Metakaolin Geopolymer Samples (HS and HS60)

Hybrid polysiloxane–geopolymer samples were prepared by incorporating 10% by weight of a commercial oligomeric dimethylsiloxane mixture into the freshly prepared metakaolin-based geopolymer suspension under mechanical stirring, when the polycondensation reaction of both the geopolymer and dimethylsiloxane had already started but were far from completion. Moreover, in this case, the photocatalyst (3% by weight) was added to the freshly prepared polysiloxane–geopolymer paste and quickly incorporated by controlled mixing (5 min at 1000 rpm).

#### 2.2.3. Foamed Hybrid Siloxane–Metakaolin Geopolymer Samples (FHS and FHS60)

Hybrid polysiloxane–geopolymer samples were prepared as described in Section 2.2.2. Afterwards, the photocatalyst (3% by weight with respect to the geopolymer paste) was added to the freshly prepared geopolymer composite paste and quickly incorporated by controlled mixing (5 min at 1000 rpm). Finally, silicon powder (0.03% by weight) was added as a foaming agent, and the system was mixed for a further 5 min at 1000 rpm. In this way, an inorganic foaming process can be induced thanks to the gas evolution (hydrogen) during the consolidation of the geopolymer mixture, as reported in the literature [34].

#### 2.2.4. Curing Treatments

As soon as prepared, an initial set of metakaolin- and fly-ash-based specimens (MK; HS; FA) was cast in the Petri dishes and cured in >95% relative humidity conditions at room temperature for 7 days and left for another 21 days in air at room temperature. A second set of samples (MK60; HS60; FA60) was cast in glass Petri dishes and cured in the same relative humidity conditions at 60 °C for 24 h and then kept still in >95% relative humidity conditions at room temperature for another 6 days. Afterwards, the specimens were kept for another 21 days in air at room temperature.

A different curing treatment was reserved for the foamed siloxane–metakaolin-based photocatalytic samples: an initial set of specimens (FHS) was cast in the Petri dishes and cured in >95% relative humidity conditions at room temperature for 7 days and left for another 21 days in air at room temperature. A second set (FHS60) of samples was cast in glass Petri dishes and cured in the same relative humidity conditions at room temperature for 24 h and then at 60 °C for another 24 h. Afterwards, the specimens were kept still in >95% relative humidity conditions at room temperature for another 6 days, and kept for another 21 days in air at room temperature.

All the metakaolin-based samples started solidifying in a few minutes. At the same time, while FA specimens presented a setting time of about 12 h, FA60 samples started solidifying within about 8 h.

#### 2.2.5. Cement-Based Reference Sample (OPC)

A reference photocatalytic cement paste sample was prepared as follows: 5.40 g of titanium dioxide (P25) were suspended in 60 g of deionized water, and 120 g of white Portland cement powder (chloride content 0.02%, *w*/*w*, sulfate content expressed as SO_3_ 2.49%, *w*/*w*) were then added. The paste was mechanically mixed using the following procedure: 60 s at low speed, 30 s at high speed, 90 s pause with no mixing, and finally 60 s at high speed. The paste was then poured in the Petri dish and treated with 30 flow table cycles. The sample was allowed to settle for 7 days into a curing chamber (20 °C, >90% RH) and then equilibrated into an environmental chamber (23 °C, 50% RH) until constant weight was achieved. During settling and weight equilibration, the sample was exposed in dark conditions to unfiltered laboratory air. The titanium dioxide content of the sample OPC is 3% (as a titania–cement paste weight ratio). 

### 2.3. SEM Analysis

SEM analysis was carried out by means of a Phenom Pro X Microscope (Phenom-World B.V., Eindhoven, The Netherlands) on the surface and fracture surfaces of the samples, without further treatments. The acceleration voltage was in the range 5–15 kV. The energy dispersive X-ray spectrometer (Phenom-World B.V., Eindhoven, The Netherlands) has the following specifications: silicon drift detector, thermoelectrically cooled (LN_2_ free); the X-ray window has ultra-thin silicon nitride (Si_3_N_4_) operating with Mn Kα ≤ 137 eV energy resolution. EDS (Phenom-World B.V., Eindhoven, The Netherlands) analyses were carried out both on the surface and at different depths, along the section of each sample. The corresponding titanium content (see Table 3) was reported as the average of the four samples.

### 2.4. Apparent Density and Open Porosity Determination

The hydrostatic weighing technique for apparent density and open porosity measurements was carried out by means of a balance OHAUS-PA213 provided by Pioneer. The samples were dried in an oven at 110 °C for 12 h and weighed after cooling at room temperature (weight of dry sample: *m*_d_). Afterwards, the specimens were placed in an empty desiccator and kept in a vacuum for 30 min. Later, the desiccator was filled with water, and the samples were kept immersed for 2 h in a vacuum and then weighed (weight of soaked sample: *m*_s_). Finally, the samples were weighed when immersed in water at atmosphere pressure (soaked immersed sample: *m*_i_). Apparent density (*D*) and open porosity (*P*) can be expressed according to the following:

(1) $$D=\frac{m_{d}}{m_{s}-m_{i}};$$

(2) $$P=\frac{m_{s}-m_{d}}{m_{s}-m_{i}},$$

### 2.5. Photocatalytic Activity Characterization

The photocatalytic activities were measured with a dedicated experimental system based on a previously described apparatus for the measurement of the photocatalytic degradation of volatile organic compounds [35].

**Figure 1:**
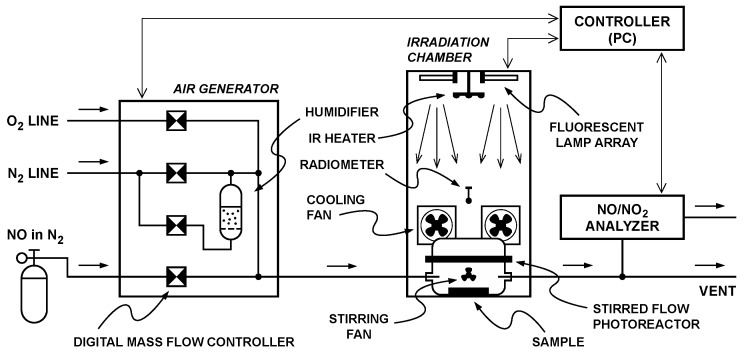
Experimental system for the measurement of the photocatalytic degradation of NO at ambient conditions and a constant NO concentration.

Briefly, the computer-controlled system (Figure 1) comprises an air generator based on digital mass-flow controllers (model 5850S, Brooks Instrument, Hatfield, PA, USA), a stirred flow photochemical reactor installed inside an irradiation chamber, and a chemiluminescence NO/NO_2_ analyzer (model 200E, Teledyne, San Diego, CA, USA). The stirred flow photoreactor ensures the uniform reactant concentrations at the sample surface even at a high conversion factor. This allows for the avoidance of both the errors due to the longitudinal concentration gradient on the catalyst surface (that is characteristic of laminar flow reactors) and the error propagation in the calculated reaction rate due to a low conversion operation (i.e., differential conditions).

**Figure 2:**
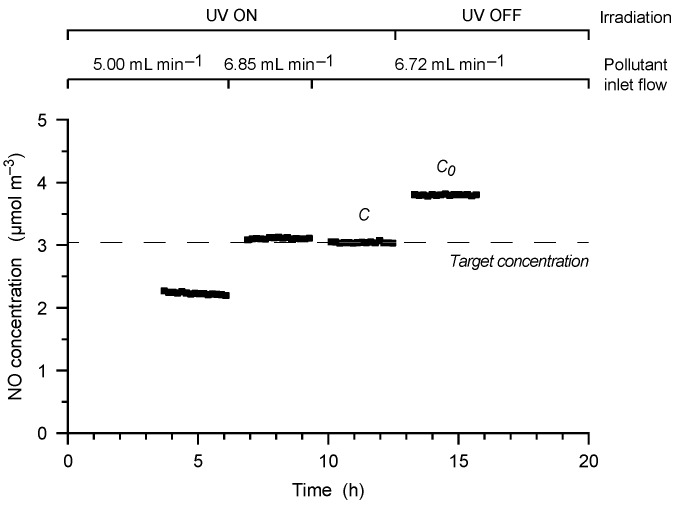
Catalytic activity measurement with the successive approximation process. At the third iteration the pollutant inlet flow required to reach the target concentration *C* is found; the UV source is then turned off, and the concentration *C*_0_ is measured.

The sample photocatalytic activity can be expressed as degradation rate according to the following:

(3) $$r=\left(C_{0}-C\right)\frac{Q}{A}$$

where *r* is the degradation rate (mol·m^−2^·s^−1^), *C* and *C*_0_ are the equilibrated photoreactor pollutant concentrations with and without irradiation respectively (mol·m^−3^), *Q* is the photoreactor volumetric air flow rate (m^3^·s^−1^), and *A* is the exposed sample area (m^2^). In order to ensure that all the measurements were carried out at the predefined NO concentration independently from the sample activity, a specifically developed constant-concentration analytical method was used [35]. This method works to reach the desired reactor internal NO concentration (under UV irradiation) by modulating the inlet pollutant flow in a successive approximation trial (Figure 2). After the reaching and the confirmation of the desired target concentration, the UV source is turned off, and the concentration in dark conditions is measured after equilibration. 

This is particularly important in the case of the comparison of samples with very different activities because, according to (3), operating with a fixed inlet NO concentration will result in very different internal reactor concentrations and, consequently, in reaction rate values measured at substantially different conditions. The use of a flow photoreactor works to take all the concentration measurements in steady-state conditions following the equilibration of the sample with the target pollutant in the reactor internal atmosphere.

## 3. Results and Discussion

### 3.1. Physical Characterization

A wide set of photocatalytic AAM samples was prepared incorporating a commercial titanium dioxide photocatalyst into several matrices with various compositions. Mix design and curing conditions are recalled in Table 2.

Foamed samples (FHS and FHS60) showed a relatively low apparent density, with values equal to 0.83 and 0.71 g·cm^−3^ for room temperature and 60 °C curing, respectively. For other samples, apparent density ranged from 1.25 to 1.67 g·cm^−3^. The low open porosity value of hybrid samples (HS and HS60) indicates a potential low accessibility of photocatalyst by reacting species. 

### 3.2. Photocatalytic Activity

The photocatalytic activity was studied for all samples measuring the NO degradation in air at ambient concentration. For all measurements, both the NO and NO*_x_* degradation rates were reported (NO*_x_* rate *r* is calculated as the algebraic sum of NO and NO_2_ values).

In order to measure the catalytic activity in consistent conditions throughout the study, all measurements were carried out operating at constant NO concentration as previously described (i.e., the NO concentration *C* inside the irradiated reactor was the same for all samples within ±3% tolerance).

**Figure 3:**
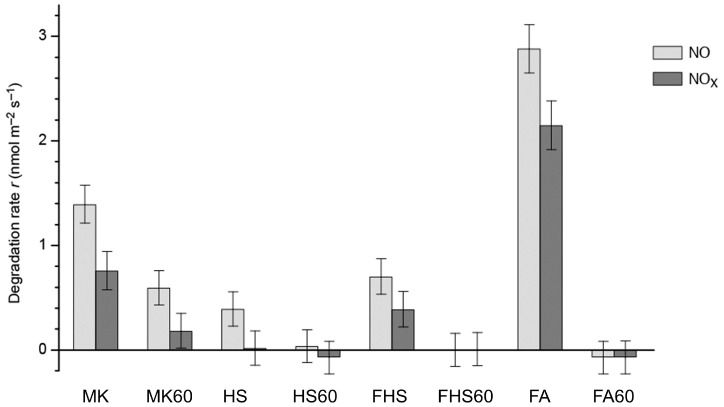
NO and NO*_x_* degradation rate *r* for the alkali activated material (AAM) photocatalytic samples. Measures carried out at 75 ppb NO concentration and 400 ± 10 μW·cm^−2^ UV-A irradiance. Bars are 1 σ repeatability errors.

The obtained photocatalytic activities of the AAM samples were reported in Figure 3. These measurements were carried out using ambient NO concentration (75 ppb nominal, 3.045 μmol·m^−3^ at 27 °C, 1 atm) at 27 ± 0.2 °C, 50% ± 5% RH and 700 ± 10 mL·min^−1^ air inlet flow. The 400 ± 10 μW·cm^−2^ UV-A irradiance was obtained with four 9-W Philips PL-S/10 UV-A fluorescent lamps (all errors are 1 σ estimated repeatability). Before the activity measurement, the samples were equilibrated for more than 30 days in dark conditions at 23 °C, 50% RH. 

The samples demonstrate very differentiated photocatalytic activities depending on aluminosilicate source and on curing conditions, with NO degradation rate values spanning from about 3 nmol·m^−2^·s^−1^ to zero (no measurable activity). All samples demonstrate lower NO*_x_* degradation rate in comparison to the NO value, indicating that the NO oxidation was not complete and in these conditions, some NO_2_ was desorbed from the samples before mineralization. All the samples cured at 60 °C demonstrate a remarkably lower activity than the corresponding samples cured at RT. In some cases, the samples cured at 60 °C does not demonstrate any measurable NO degradation activity. The sample FA based on fly ash and cured at RT demonstrates the best performance with a twofold NO degradation rate compared with the metakaolin-based sample MK. The hybrid sample (HS) demonstrates a remarkable smaller catalytic activity (about 30% of the MK sample NO degradation rate). 

**Figure 4:**
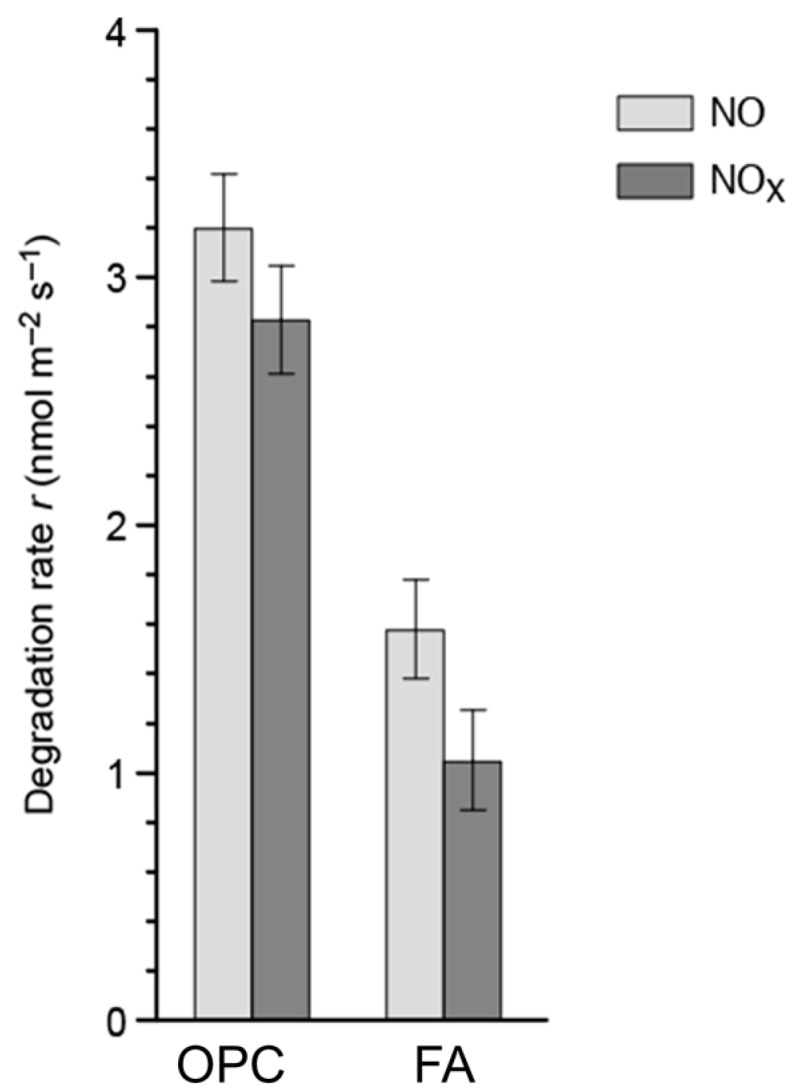
NO and NO*_x_* degradation rate *r* for the ordinary Portland cement photocatalytic sample (OPC) and for the fly ash AAM sample (FA). Measures carried out at 75 ppb NO concentration and 120 ± 5 μW·cm^−2^ UV-A irradiance. Bars are 1 σ repeatability errors.

The further addition of an expanding agent (metallic silicon) on sample FHS shows appreciable improvement, but the activity of this sample is lower than that of the metakaolin sample MK. The sample FA activity (Figure 4) was then compared with the ordinary Portland cement reference sample OPC using a lower irradiance (120 ± 5 μW·cm^−2^ UV-A) in order to avoid an excessive conversion rate for the latter sample. The photocatalytic activity of the sample FA is significantly lower than the activity of the reference sample OPC, but it can nevertheless be considered in the same order of magnitude (about a factor two difference). This result is particularly interesting considering the room for further optimization given by the characteristic variety of AAM materials and the highly differentiated photocatalytic activities demonstrated in the present work. 

### 3.3. Scanning Electron Microscopy Analysis

**Figure 5:**
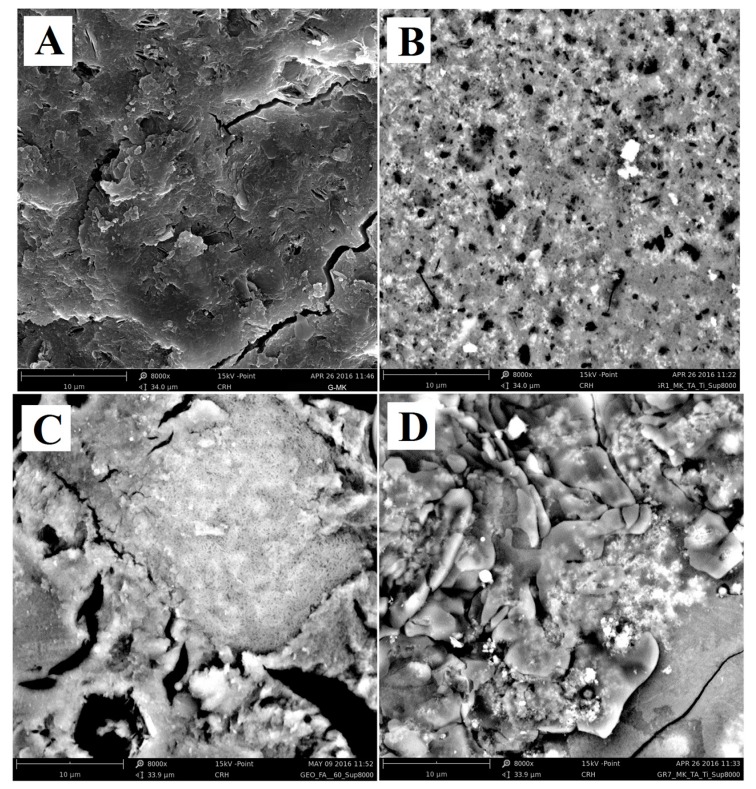
SEM images of surface at 8000 magnifications of the samples: (**A**) metakaolin-based geopolymer; (**B**) metakaolin (MK); (**C**) fly ash-based geopolymer; and (**D**) FA.

Because of UV radiation penetration and the reactant diffusion limits, the heterogeneous photocatalytic degradation of airborne pollutant is governed by surface processes. In order to study the catalyst distribution in the AAM matrices, a series of SEM analyses was carried out on the surfaces of AAM specimens that showed the most interesting photocatalytic activities (MK and FA samples). Particularly, the SEM images of the surface of the examined samples were reported in Figure 5. This figure shows that a pristine (i.e., without photocatalyst) metakaolin-based geopolymer sample (Figure 5A) is characterized by a compact morphology revealing some unreacted kaolinite crystals. The sample MK (Figure 5B) shows a lesser compact surface structure when compared with the pristine geopolymer, with the presence of pores of a different size, uniformly distributed. 

The pristine fly-ash-based geopolymer (Figure 5C) is characterized by a very disaggregated morphology, typical of this kind of geopolymers [36,37,38]. The specimen FA (Figure 5D) shows a complex morphology where it is not possible to clearly identify the presence of pores, but the surface structure appears rather uneven with the presence of small domains, and some appear spheroidal.

**Figure 6:**
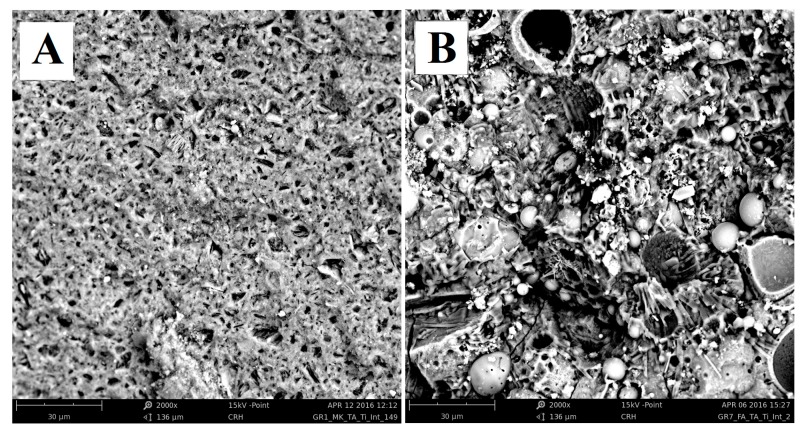
SEM images of internal section at 2000 magnifications of (**A**) MK and (**B**) FA samples.

SEM images of sections of the MK and FA samples have been also carried out (Figure 6). While the metakaolin-based sample MK (Figure 6A) shows a poorly compact morphology, quite similar to that one analyzed on surface of the sample, the morphology of the FA sample (Figure 6B) is dominated by the presence of unreacted fly ash particles that are well dispersed in the geopolymer matrix. The uneven morphology of the FA sample is due to the limited reactivity of the fly ash particles and causes the non-completeness of the geopolymerization reaction.

In order to assess the titania distribution on the sample, the relative titanium content on the surface and at different depths along the specimen section was measured with SEM/EDS (Table 3).

The obtained data indicate uneven catalyst distribution between the surface and the initial layers (up to 600 μm of depth) of the inorganic matrices. Particularly, the fly-ash-based sample cured at room temperature shows higher surface titanium content than metakaolin-based sample cured in the same condition. In addition, both samples cured at 60 °C indicate a surface Ti content lower than the corresponding samples cured at room temperature. Segregation phenomena of titania can be caused by several physico-chemical phenomena, including agglomeration determined by low shear mixing or by particle-to-particle surface interactions. The data suggest a possible convective transport during the casting and curing phase where surface water evaporation can drive local redistribution of unreactive low dimension particles with marked dependence on ambient conditions (e.g., temperature and RH). It is worth pointing out that the samples that show a higher surface amount of TiO_2_ also possess a higher catalytic activity. Meanwhile, the samples that have a lower concentration of catalyst on the surface show evident segregation phenomena of TiO_2_ in depth (i.e., MK60 and FA60 samples, see Table 3). Moreover, all the studied samples shows a marked decrease in the photocatalytic activity if cured at 60 °C (Section 3.2), and this appears to be reflected by a significant decrease in the surface titania content in the corresponding analyzed sections. These data suggest that titania distribution in the sample surface layers can play a relevant role in the determination of the final sample photocatalytic activity.

## 4. Conclusions

An initial comparative assessment of the AAM binders potentials as photocatalyst support matrices was carried out using four different types of AAM: metakaolin geopolymer; fly ash geopolymer; hybrid siloxane–metakaolin geopolymer; and foamed hybrid siloxane–metakaolin geopolymer. The samples was characterized by means of SEM-EDS analysis. 

The photocatalytic activity of the samples was evaluated in terms of NO abatement. The photocatalytic activity data show a strong variation depending on the type of binder and the curing process. The highest photocatalytic activity was detected for fly ash-based AAM matrices cured at room temperature. Metakaolin-based AAM matrices also showed promising photocatalytic activity. 

A systematic decrease of photocatalytic activity was observed when the same AAM support matrix was cured at higher temperature (60 °C).

EDS data of the studied samples indicate a conspicuous segregation effect depending on AAM matrix and curing temperature, with a notable depletion of the surface titania content for the samples cured at 60 °C. Photocatalytic activity data correlate with the surface titania content measurements suggesting that titania segregation may play a distinct role in the determination of photocatalytic activity.

The described results demonstrate that AAM binders can be interesting photocatalyst support matrices. The high variation of catalytic activity evidenced by the different samples and the inherent variety of AAM binders suggest, moreover, large possibilities in performance enhancement. Particularly, the described results indicate that the optimization of the photocatalyst dispersion by the curing process tailoring and selection of the AAM aluminosilicate precursor–activating solution combination can play a fundamental role in the development of high performance AAM photocatalytic materials. Furthermore, given the good activity of the studied AAM samples in comparison to ordinary Portland cement matrix it is reasonable to expect the future development of high performance photocatalytic AAM materials. 

## Figures and Tables

**Table 1:** Chemical composition (wt %) of the fly ash, metakaolin, and sodium silicate solution used in this paper.

**Fly Ash**							
Al_2_O_3_	SiO_2_	K_2_O	Fe_2_O_3_	Na_2_O	MgO	CaO	others
21.71	48.59	2.11	8.03	1.06	2.40	7.32	8.78
**Metakaolin**							
Al_2_O_3_	SiO_2_	K_2_O	Fe_2_O_3_	TiO_2_	MgO	CaO	others
41.90	52.90	0.77	1.60	1.80	0.19	0.17	0.67
**Sodium Silicate Solution**							
SiO_2_	Na_2_O	H_2_O	-	-	-	-	-
27.40	8.15	64.45	-	-	-	-	-

**Table 2:** Composition (wt %), curing conditions, apparent density, and open porosity of the samples used in this study. Photocatalyst (3% *w*/*w*) was added to all formulations immediately after preparation.

Sample	Mk	Fa	SS	NaOH	NaOH soln	Resin	Si	Curing	Open Porosity (%)	Apparent Density (g·cm^−3^)
MK	41.6	-	50.0	8.4	-	-	-	RT	39.74	1.46
MK60	41.6	-	50.0	8.4	-	-	-	60 °C, 24 h	38.89	1.40
HS	37.4	-	45.0	7.6	-	10	-	RT	13.96	1.36
HS60	37.4	-	45.0	7.6	-	10	-	60 °C, 24 h	12.66	1.25
FHS	37.4	-	45.0	7.6	-	10	0.03	RT	34.71	0.83
FHS60	37.4	-	45.0	7.6	-	10	0.03	60 °C, 24 h	53.16	0.71
FA	-	66.2	24.4	-	9.4	-	-	RT	28.80	1.67
FA60	-	66.2	24.4	-	9.4	-	-	60 °C, 24 h	37.74	1.48

Mk = metakaolin; Fa = fly ash; SS = sodium silicate solution; NaOH = sodium hydroxide; NaOH soln = aqueous sodium hydroxide solution 10 M; Resin = silicone rubber; Si = metallic silicon powder (evolves H_2_ during curing).

**Table 3:** Relative titanium content for selected samples (energy dispersive X-ray spectrometry (EDS) analyses).

Sample	Ti Surface (%)	Ti 150 μm (%)	Ti 300 μm (%)	Ti 600 μm (%)
MK	2.8 ± 0.1	4.8 ± 0.2	8.6 ± 0.2	7.7 ± 0.2
MK60	1.7 ± 0.2	1.8 ± 0.2	2.8 ± 0.1	2.9 ± 0.1
FA	4.0 ± 0.2	0.8 ± 0.1	1.2 ± 0.1	1.5 ± 0.1
FA60	2.6 ± 0.1	2.1 ± 0.1	7.9 ± 0.2	4.2 ± 0.2

## Abbreviations

The following abbreviations are used in this manuscript:

AAM	alkali activated material
PCO	photocatalytic oxidation
EDS	energy dispersive X-ray spectrometry
RT	room temperature
RH	relative humidity
